# Supplementary material for: Antimicrobial and Antibiofilm Effect of 4,4′-Dihydroxy-azobenzene against Clinically Resistant Staphylococci
Source: Antibiotics (Basel). 2022 Dec 11;11(12):1800. doi: 10.3390/antibiotics11121800 (PMC9774766; doi:10.3390/antibiotics11121800)
Supplement: Supplementary file 1 [file antibiotics-11-01800-s001.zip › antibiotics-2061048-supplementary.pdf]

Pérez-Aranda et al. Supplementary information Figure S1

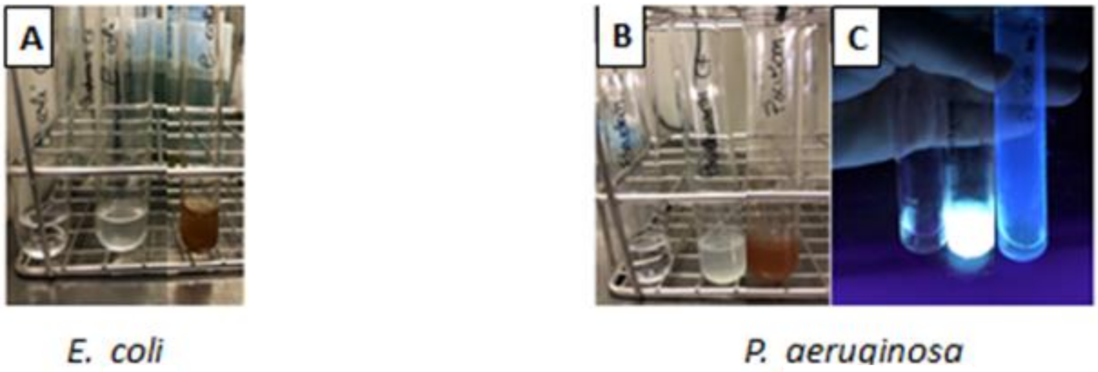

Strain	Negative control (minimal medium without any C source)	Positive control (TSB)	Minimal medium containing DHAB (512 mg L^{-1})
Escherichia coli **ATCC 25922**	1.3 x 10^5 CFU.mL^1	4.1 x 10^8 CFU.mL^{-1}	1.8 x 10^7 CFU.mL^{-1}
Pseudomonas aeruginosa **ATCC 27853**	3.3 x 10^5 CFU.mL^{-1}	Confluent growth	9.2 x 10^6 CFU.mL^{-1}

Supplementary information Figure S1: Growth of *E. coli* ATCC 25922 and *P. aeruginosa* ATCC 27853 in the presence of DHAB as the sole C source.

A. *E. coli*, from left to rigth, grown in minmal medium without C source, TSB and minimal medium with DHAB (512 mg L^{-1}).

B *Idem* for *P. aeruginosa*.

C: *P. aeruginosa* cultures observed under UV light.

In the table, data of colonies forming units (CFU mL^{-1}) from tubes in the pictures (average of three replicates).

Pérez-Aranda et al., Supplementary Information Figure S2

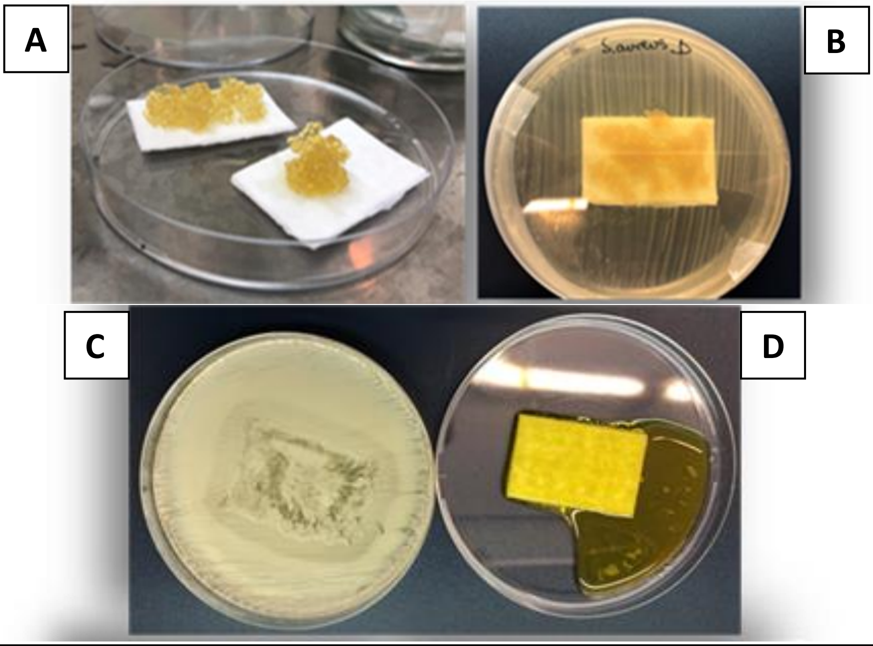

Supplementary information Figure S2. Different carriers for application of DHAB. A, B: Preparation of alginate beds containing DHAB. C: Result of the application of DHAB alginate beds, showing weak diffusion of the substance to the medium. D: Result of the preparation of a composite containing gelatine and DHAB. The bacteria were able to degrade gelatine and the composite was not stable.
